# Supplementary material for: Impact of the COVID-19 pandemic on the mental health and well-being of adults with mental health conditions in the UK: a qualitative interview study
Source: J Ment Health. 2021 Jul 29;32(6):1040–7. doi: 10.1080/09638237.2021.1952953 (PMC12094262; doi:10.1080/09638237.2021.1952953)
Supplement: Supplemental Material [file IJMH_A_1952953_SM6780.docx]

**Supplementary Table 1. Themes and supplementary supporting quotes**

| **Themes** | **Supporting Quotes** |
| --- | --- |
| *Factors contributing to a deterioration in mental health* | |
| 1. Feeling safe but isolated at home | *Just the loneliness has brought me down, really. I live on my own. It does have its good points, but I have noticed that sometimes, I do get down. Just mulling over things, just because I’m living on my own (ID20).*  *Well, I am running out of things that I would normally get from shops in the town. I don’t want to go to the town at all, I’m frightened of it (ID05).*  *Although I was quite shocked by how quickly everything was lifted and it did frighten me, and I stopped going out, the first two days after it was lifted because I was frightened by how quickly people were getting pushy in the supermarkets (ID04).*  *I’ve hardly gone out. I suppose most days I’ve been in, and I feel safe indoors, and that’s where I intend to stay until I feel safe to go out, regardless of what anybody is telling me that it’s safe out there. Unless I believe it, I just don’t want to. And my anxiety about the outside world is so extreme now. I’d only just got confident in going out (ID05).*  *Being physically on my own a lot more has been difficult, so there have been times when the overwhelming feelings that I can generally hold at bay have been crashing in. It hasn’t been loads of times, but it has been sometimes (ID23).* |
| 1. Disruption to mental health services 2. Disruption to mental health services | *I struggled at the beginning of the lockdown with more anxiety and depression and mentioned it to the nurse at the surgery. Their response was that, even if I did need to see someone, there was no one virtually to refer me to anyway. So, it hasn’t felt as though, if I’d needed support in that way, that there would have been any available really from the health service (ID23).*  *I tried to contact the crisis line who were unhelpful towards me and they told me to speak to my GP about getting my meds changed. So, I rang up the GP and they rang me back and I got a trainee GP who had the nerve to ask me why I did that and they didn’t know why I would do something like that deliberately. And then he suggested I get back in touch with the crisis line, so they were effectively sending me round in circles (ID12).*  *I’m seeing my care coordinator less. I only see her at clinics now. Other than that, she doesn’t come around, their direction is not to see people in their homes at the moment…* *I haven’t felt that way but I’m aware that sometimes when I don’t feel I need it, I do need it (ID25).*  *They (Samaritans) put me at ease. Yes, they couldn’t allay my fears or anything because everybody’s going through the same thing, but they were able to calm me down and talk rationally to me. I rationalised things and came off the phone, feeling a lot different (ID05).*  *So, I was worried that there would be more of an impact, basically more people unwell, so those, in a way, who are already unwell, basically they’ll be, like, we can’t look after all of you, and that people are going to be shut out of the system (ID16).*  *It’s the depression that I’ve had the problems with because the loneliness has just brought on another episode. …That’s why I’ve had to have a consultation with a GP, and the lack of face to face was really apparent, I almost felt she was a bit flippant. It’s somebody I hadn’t met before, so that didn’t help (ID22).*  *I don’t even know what [name] is, but he’s always there. He actually had a word with me yesterday, he called to check on me to see how am I. So, I’m really pleased for that support that I have in that sense…* *if I’m not with these people, where is the support going to come from? (ID14).*  *I was part of a group, it was for three months, I did eight weeks on it, maybe less. But then lockdown happened and then said they couldn’t see anyone face to face and they set everything to go online. But I didn’t find that helpful, the online one, so I withdrew from the course… I’d find myself sitting for the entire session, two hours, not saying a word, where there were people that put a lot of input into it. There wasn’t a lot of room for many other people to take part (ID25).*  *…and so a sense that if it’s not urgent, don’t contact your GP. No one ever said that but that was the sense I got, so it almost felt like my depression was not something that is a priority at the moment and it still feels that way. Never mind not feeling like they’d be able to give me the kind of support that I’m looking for anyway and I know I’d just go on medication without the counselling… (ID10).* |
| 1. Cancelled plans and changed routines | *It’s meant the things that I had planned to do with people I wasn’t able to do. For example at the end of March I wasn’t able to have the celebration tea party I’d planned to have, I had to cancel that. I don’t know when we’ll be able to have it (ID23).*  *We have fetes, and carnivals, and stuff like that in our community, and they’re cancelled this year, and the thing is, when are we going to ever do those? But also, when are people going to feel safe to attend them, because even if people decide to put on an event, that’s not to say that people will attend, and this could go on for years, couldn’t it? (ID05).*  *My friend [name] suggested for New Year we should rent out a house so we’ve got that on the go. And then my other housemate they had a wedding but they just signed some paperwork. So, they’re meant to be organising their actual wedding party next year… So, yes, I think that’s keeping me going (ID11).*  *I think it’s difficult to establish a routine, and having a routine is important, because sleeping particularly, that can cause bipolar symptoms to flare up, depression, it’s a very fine balance …..That was a big anxiety, that I was probably getting up about more nine o’clock-ish, and that was a huge anxiety for me. I like to have a routine, it’s really important to me (ID05).*  *This rumination that I think it’s called, where you go inward and there’s a dialogue going in your brain, I wonder if I’m going to get ill, I wonder if I get a new illness, I wonder if I need a doctor and I can’t get seen. All of these things start accumulating in your head, where in normal routine, in daily life, it’s so busy those things get buried beneath the busyness, but they’ve certainly come to the fore during this crisis for me, they really have (ID22).* |
| 1. Uncertainty and lack of control 2. Uncertainty and lack of control | *It was a really, really difficult time. And it all happened in such a short space of time. It was like you couldn't imagine another thing happening and it happened, and then it just got worse and worse (ID15).*  (*Anxiety) was sky-high to begin with, because I didn’t know what was going on, and I was in panic mode (ID08).*  *I had a lot of anxiety when it was coming up to the review about whether the government would ease the lockdown, and I didn’t want to go out, I didn’t want to be forced to go out (ID05).*  *I do feel like, in all of this, there was very little that we could actually control and I think that was quite an uncomfortable place to be in, where the things you can control are really tiny (ID06).* |
| 1. Rolling media coverage | *I noticed the days when I focussed on that all day were bad days. And the days when I managed to switch that off for quite a few hours and I would still watch one bulletin in the evening, those were better days, so I’ve tried to stick to that since then (ID18).*  *None of us have been in this situation before, I don’t know how to get perspective on it sometimes. I’ve tried not to think about the global position too much, and I don’t look at the news all the time, I maybe only do it once or twice a day to get information (ID05)*.  *Initially, yes, it was pretty bad, and it’s not helped by the fact you’re awake at two o’clock in the morning, so you switch the radio on, just to send you back to sleep, when all they’re going on about… Yes, I’d say that was another negative, is the blanket news. Okay, there isn’t much else going on, but oh boy, you don’t have to say the same thing 120 times (ID02).*  *I read the information in the news, people were dying as a result, I couldn’t seem to summarise in my mind to what was actually going on and what had happened, I was too ill (ID19).* |
| *Coping strategies and protective factors* | |
| 1. Previous experience of adversity | *I don’t know how some people would have managed, but I’m strong enough, I’m will-powered enough I’ve learned during actions I’ve undertaken in just enabling myself to better live with a condition. I’m managing through the lockdown period (ID19).*  *I have gone through periods where I am isolated and agoraphobic, so lockdown wasn’t the first time I’ve had my own personal lockdown (ID25).* |
| 1. Feeling less accountable to others | *I love the lack of responsibility; I love not being accountable to anybody. Silly things like, I’ve haven’t dyed my hair since whenever, so it’s nearly white now. I don’t give a, you know. I haven’t put make up on, because nobody’s going to see me. It makes you wonder why you do some things (ID02).*  *I think I’ve been a lot more worried about identity, than I was aware of being, and that sort of conforming to and being acceptable to other people, and that doesn’t matter at the moment (ID02).*  *There is a sort of peace in knowing that you’re not under pressure to do anything other than go for a walk in the park and get through the day, and do a bit of reading or writing, or whatever (ID18).*  *I do think the lockdown’s made things slightly easier for me because more people have had to stay in and it’s been nice to be able to compare my life to others where they’ve not been able to go out and do whatever…. Not having people try to go at me because I haven’t been able to get about. That’s been really nice. And not to have the feeling of I should go out but I can’t get out of the door. That is a real big one to think I shouldn’t go out, it’s not that I don’t want to, I actually shouldn’t. That has been really nice, that sounds weird but I’m not great at going out (ID25).*  *Also, fewer people around means, I just feel a little bit freer. Like, by default, I would spent quite a lot of time talking to myself or just singing, you know and previously would have either tried to not do that or put a limit on it when I was out in the world. I felt quite self-conscious about it or whatever, but nowadays I can do it without feeling particularly self-conscious or anything like that because there’s not many people around (ID24).*  *To start with, my anxiety, it came down some pegs, because the whole world was feeling the same way about germs and contamination that I’ve had for most of my life, those anxieties. I hate the word normal, but I felt like everybody else, and I think other people have said that they’ve felt like in their whole life they’ve been preparing for this situation (ID05).* |
| 1. Engagement in hobbies and activities 2. Engagement in hobbies and activities | *Doing so many different creative things, there’s no reason why I shouldn’t have done them before, but I’m not going to get interrupted by going to a meeting or going out with friends or anything. My house can be complete chaos, there’s lots of creative stuff all over the place, and I can just play. All play (ID02).*  *I had a lot of canvas board and paints that were unopened. Again, things I’ve always thought I’ll do at some point, so I’ve done quite a bit of painting. They’re not great, but still, good time (ID08).*  *Just revisiting hobbies. The timing was quite lucky actually, just before the pandemic arrived, I bought a sewing machine so I’m revisiting my sewing hobby, which is lucky I got it when I did, because if I hadn’t bought the machine in time, I would have struggled to get it. Yes, I’ve had a bit more time…which I’ve tried to use in a positive way. An enjoyable way (ID24).*  *I have been doing some online Zoom courses with a, I don’t know if I can say it, it’s a [location] drawing organisation, and they do online creative sessions, they’re called a pay as you go, or pay as you feel…They’re only like an hour generally, and there are people all over the world on them, and all over the country, they’re fantastic. They’re an hour, and they’re completely absorbing, I just draw, do creative stuff.*  *For me, I think that’s the most mindful thing I suppose I do all week now, is that group (ID05).*  *I’ve enjoyed all the extra things I’ve done, all the reading, language learning, exercise, cooking, I’ve been baking (ID12).* |
| 1. Staying connected with others 2. Staying connected with others | *I guess the fact that I did have a number of existing networks has helped, so I’ve been able to tap into those I guess. When we’ve had some of the Zoom meetings, just done a quiz or things like that that have not been anything to do with the pandemic or directly giving support to each other but has taken your mind off it. To just be able to do things over the phone or video call like that to take your mind off everything else. (ID23)*  *Those old friends that you’d taken for granted, or didn’t seem particularly relevant any more, to realise that if that connection can be rekindled after such a long time, meaningfully, it was worthwhile. So, it’s going to be worth making more effort about in the future (ID02).*  *I haven’t seen them in quite a few months now. I’ve messaged a few of them, but it’s not the same as seeing them in person. Some of them are not messaging, I feel like they don’t prefer to message, but seeing, meeting up in person (ID17).*  *I think, in terms of social groups for positive things, my book club, we’ve still been trying to have our book club meetings on Zoom, where we’d normally go out for dinner. And it’s definitely not as easy, because trying to chat with eight or nine people all talking at once can be quite difficult, but it’s nice still to have that engagement with them (ID06).*  *What’s really, really pleased me, emotionally, instead of spending more time online chatting and chatting and chatting, we just sit as a family and we chat. My son spent two hours with his friends chatting. Come back again as a family, we chat (ID14).* |
| 1. Perceived social support | *I tend to, when I isolate, I get ill. So, I’ve been staying at my mum’s for the last few weeks, they were noticing some downhill factors when I’ve been in my flat for the entirety of lockdown (ID25).*  *I wouldn’t say we know a lot of our neighbours but we certainly chat with a lot of our neighbours and we’ve traded numbers with people and offered our help because I’m going shopping, weekly mostly. We’ve traded numbers and I’ve said I would happily get shopping or whatever it might be, but maybe, actually a little bit surprisingly, nobody has taken us up on that and we’ve not needed the support of that kind ourselves (ID24).*  *I am quite an active person in my community, at the beginning there was a huge amount of pressure, it felt, on anyone of an active kind of age to maybe be going out and about delivering supplies, prescriptions, all that kind of thing. I think that was from the very, very early days, so I suppose I felt a lot of guilt about not doing more for other people (ID05).*  *I’ve found a voluntary activity I can do which is working at a food bank food kitchen. I go into town and I do that once a week, which is good for helping me feel like I’m doing something positive for the community (ID12).*  *The friend that I help who’s got dementia has had to be via Zoom or phone calls, so that’s been frustrating. I feel that I can’t help her as much as I want to help her because I can’t go in the house… (ID23).* |
